# Glioblastoma multiforme restructures the topological connectivity of cerebrovascular networks

**DOI:** 10.1038/s41598-019-47567-w

**Published:** 2019-08-13

**Authors:** Artur Hahn, Julia Bode, Thomas Krüwel, Gergely Solecki, Sabine Heiland, Martin Bendszus, Björn Tews, Frank Winkler, Michael O. Breckwoldt, Felix T. Kurz

**Affiliations:** 1 Heidelberg University Hospital, Department of Neuroradiology, Heidelberg, 69120 Germany; 2 University of Heidelberg, Department of Physics and Astronomy, Heidelberg, 69120 Germany; 3 University of Heidelberg and German Cancer Research Center (DKFZ), Schaller Research Group, Molecular Mechanisms of Tumor Invasion, Heidelberg, 69120 Germany; 4 Heidelberg University Hospital, Neurology Clinic and National Center for Tumor Diseases, Heidelberg, 69120 Germany; 5 German Cancer Consortium (DKTK), Clinical Cooperation Unit Neurooncology, Heidelberg, 69120 Germany; 6 German Cancer Research Center (DKFZ), Clinical Cooperation Unit Neuroimmunology and Brain Tumor Immunology, Heidelberg, 69120 Germany

**Keywords:** Cancer models, CNS cancer, Preclinical research, Computational biophysics, Network topology

## Abstract

Glioblastoma multiforme alters healthy tissue vasculature by inducing angiogenesis and vascular remodeling. To fully comprehend the structural and functional properties of the resulting vascular network, it needs to be studied collectively by considering both geometric and topological properties. Utilizing Single Plane Illumination Microscopy (SPIM), the detailed capillary structure in entire healthy and tumor-bearing mouse brains could be resolved in three dimensions. At the scale of the smallest capillaries, the entire vascular systems of bulk U87- and GL261-glioblastoma xenografts, their respective cores, and healthy brain hemispheres were modeled as complex networks and quantified with fundamental topological measures. All individual vessel segments were further quantified geometrically and modular clusters were uncovered and characterized as meta-networks, facilitating an analysis of large-scale connectivity. An inclusive comparison of large tissue sections revealed that geometric properties of individual vessels were altered in glioblastoma in a relatively subtle way, with high intra- and inter-tumor heterogeneity, compared to the impact on the vessel connectivity. A network topology analysis revealed a clear decomposition of large modular structures and hierarchical network organization, while preserving most fundamental topological classifications, in both tumor models with distinct growth patterns. These results augment our understanding of cerebrovascular networks and offer a topological assessment of glioma-induced vascular remodeling. The findings may help understand the emergence of hypoxia and necrosis, and prove valuable for therapeutic interventions such as radiation or antiangiogenic therapy.

## Introduction

Vascular networks are transport networks that provide vital substances such as oxygen and nutrients to living tissue and remove biological waste products. Their characteristic morphology allows regulation of the surrounding biological environment, including thermoregulation and physiological ion balance to maintain tissue homeostasis^1^. Motivated by energy cost minimization (Murray’s law^2,3^), healthy vasculature typically follows a hierarchical arterio-venous branching scheme, with blood flowing through thick arteries, successively branching into thinner arterioles, followed by capillaries and a similarly organized venous system, draining the tissue in vice-versa^4^. Forming efficient transport networks, healthy vessel constructs are inherent to tree-structured arterial and venous parts, interwoven by dense, regular capillary beds^5–7^.

Malignant tumors disrupt the local biochemical environment and regulation of pro- and antiangiogenic factors, such as vascular endothelial growth factor, angiopoietins and Ang-2^8,9^. As a solid tumor grows, the pre-existing vasculature is adapted and constantly modified by several mechanisms including angiogenesis^10^, vessel dilation^11^, regression, constriction, and occlusion^12,13^. The complex interplay of these processes, dynamically regulated during tumor progression by biochemical, metabolic, mechanical, and hydrodynamic influences^14,15^, leads to highly heterogeneous vessel architectures throughout the tumor volume^8,16^, that may impact response to radiotherapy^17^.

Extensive research has been conducted to investigate the structure of tumor vasculature and how it sets itself apart from healthy vessel constructs. Most studies of this nature have focused on local vessel properties, such as microvascular density (*MVD*), vessel segment geometry and space-filling properties (see, *e*.*g*.^18–21^ and references therein). Although sophisticated models have been developed to analyze global properties of large-scale vessel architectures in theory^22,23^, topological analyses of vascular networks that consider properties such as local clustering of vascular nodes or inter-node connectedness remain scarce and either focus on two-dimensional vascular networks^24^, smaller three-dimensional networks, *e*.*g*., of lymph nodes^25^, or subnetworks, including only certain vessel types^26–30^.

In general, experimentally extracted vessel networks are usually constrained to either small imaging volumes or limited resolution; full vascular networks in brain tissue, including small capillaries, could only be obtained histologically or by laborious combinations of multi-scale imaging modalities, as, *e*.*g*., in^31,32^. A recent approach using fluorescence ultramicroscopy, however, made it possible to image detailed micro- and mesoscopic vascular structures of entire organs^33,34^ and was successfully applied to mice brain^35,36^. A detailed structural and functional quantification of such entire vascular networks may unveil previously unknown consequences of vascular remodeling and aid the development of targeted antiangiogenic therapies.

In this study, we present numerical quantifications of blood vessel networks from entire GL261 and U87 glioblastoma xenografts in mice, as well as comparable healthy brain regions in a mouse model, including capillaries with diameters down to approximately 3 *μ*m. Geometric properties of the vasculature, extracted from tumors and healthy brain hemispheres, were quantified using custom-written, highly scalable codes in Matlab (Mathworks, Natick, MA, USA). As in other quantitative studies of multi-scale vessel data, *e*.*g*.^37^, the fractional blood Vessel Volume (*fVV*), vessel length density ${\rho }_{L}$, and vascular surface density ${\rho }_{A}$ were determined and the individual vessel segments were characterized by their mean radius $\bar{r}$, length *l*, tortuosity $\tau$ and surface area *A*. Augmenting the geometric analysis with graph theoretical analysis tools, the vessel architectures are modeled as undirected networks to reveal local and nonlocal topological properties. The connectivity characteristics are studied on multiple length scales with the help of a network theoretical community paradigm^38^.

High local heterogeneity within and among tumors makes a global geometric characterization of tumor vasculature difficult. Nevertheless, one would expect the mechanisms of vascular remodeling during tumor growth to reflect in the global network topology of the emerging vessel constructs. The graph theoretical framework offers powerful tools for the assessment of global network characteristics of large vascular systems, encompassing 10^5^–10^6^ constituent segments. We present network theoretical quantifications on the largest cerebrovascular networks studied so far, resolve basic topological characteristics of healthy brain vasculature, and show how the vascular connectivity changes in U87 and GL261 glioblastoma.

## Methods

### Data acquisition

#### Tissue preparation and imaging

3D vessel morphology was imaged *ex vivo* using fluorescence light sheet microscopy as described before^35^. In brief, we injected $7.5\cdot {10}^{4}$ U-87MG (ATCC HTB-14) cells in 9 week old, male NOD Scid Gamma mice (NSG, DKFZ, Heidelberg) and 10^5^ GL261 glioma cells (National Cancer Institute NCI, Bethesda, MD, USA) in 6–8 week old, female C57Bl/6J mice (Charles River Laboratories, Sulzfeld, Germany; n = 6 mice). The cells were tested biweekly for mycoplasma contamination with negative outcome. Cells were diluted in 5 *μ*l sterile phosphate and buffered saline (PBS, Sigma-Aldrich Chemie GmbH, Taufkirchen, Germany) and injected in the striatum of the right hemisphere, 2 mm lateral and 2 mm ventral of the bregma. Respectively, $n=6$ animals with glioblastoma were compared against $n=6$ brain hemispheres from ${n}_{m}=3$ healthy mice as controls. All animal experiments were conducted in accordance with appropriate guidelines and approved by the regional ethics committee in Karlsruhe, Germany (permit numbers G223/14, G187/10, G188/12, G145/10, and G287/15).

21 days post tumor cell implantation for U87 specimens and 28 days post injection for GL261 mice, the animals were injected intravenously with 300 *μ*l of lectin-FITC (Sigma-Aldrich, St. Louis, MO, USA) at a concentration of 1 mg/ml. After 3 minutes of incubation, mice were sacrificed by a ketamine/xylazine overdose. Mice were transcardially perfused with 20 ml PBS and 20 ml 4% PFA. The brain was explanted and optically cleared using the FluoClearBABB protocol^34^. Upon successful tissue clearing, Selective Plane Illumination Microscropy (SPIM) was employed to image the microvasculature in the entire brain by fluorescent excitement of the lectin marker (3.25 × 3.25 *μ*m in-plane resolution and 5 *μ*m between slices in the transverse plane) with the following acquisition parameters on an Ultramicroscope II (LaVision Biotec, Bielefeld, Germany): 100% laser power, 5 *μ*m stepsize, dynamic focus on (5–10 steps), Andor camera exposure time of 686.345 ms, 16-bit low noise gain, left and right light sheet together.

#### Post-processing

The acquired image stacks were segmented using the interactive learning and segmentation toolkit ilastik^39^. To reduce noise, the binary vessel representations attained this way were smoothed with a 3D-Gaussian filter with isotropic standard deviation *σ* = 1 (voxel units), using the 3D-smoothing plugin in the ImageJ-distribution Fiji 2.0.0-rc-43/1.51r^40^. The volume was again binarized with intensity threshold at half of the maximum voxel value. A self-written script in Matlab was used to fill holes in the binary structures (i.e. “hollow” vessels) to correct for segmentation artefacts. A self-written Matlab script further reduced noise by removing isolated voxel bunches with a volume of less than a sphere with a 6 *μ*m radius (based on 6-connectivity^41^).

The skeletonization algorithm^42^ in ImageJ was used to extract the vessels’ center lines. The plugin AnalyzeSkeleton^43^ assigned a tag to each skeleton voxel, identifying end-points (with less than two neighboring skeleton voxels), junctions (with more than two neighbors), and so-called slab voxels (with exactly two neighbors). The tagged skeletons were later used for vessel/node labeling and connectivity list construction.

Binary masks were manually drawn over each image stack to select the tumor volume and corresponding regions in the healthy brain hemispheres for analysis. Care was taken that only well-resolved regions with minimal blurring were incorporated in the control datasets. This comprised the inner parts of the brain, including the midbrain, hippocampus, thalamus, hypothalamus, septum, striatum, caudate, putamen, amygdala, and inner sections of the cerebral cortex and cerebellum. The ventricular system, exhibiting false fluorescence, was blinded by the masks. Tumor boundaries were assessed visually by two neuroradiologist physicians based on microvascular anomalies (increased irregularity and overall tortuosity) in the tumor region. Tumor cores were masked separately based on a transition within the tumor vasculature from a more dense outer shell to a less vascularized center, presumed to exhibit hypoxia.

As detailed in^41^, segmentations are often ambiguous at structure boundaries, which can have great effects on vessel geometry at the given resolution. We suspect the images to be subject to “fluorescent overexposure”, which would cause background voxels to be illuminated and registered in the segmentation, causing vessels to appear thicker. To compensate for this over-fluorescence and over-segmentation, we implemented a circumferential thinning in Matlab, which eliminates the boundary layer voxels from the segmented structures, with the exception of voxels constituting the skeleton. All processing steps conducted leave the network topology unchanged by definition.

### Geometric and topological analysis

#### Vessel geometry

The masked, binary image stacks were processed in Matlab R2016b (Mathworks, Natick, MA, USA) using custom written codes. The fraction of blood-filled tissue volume marked by perfused vessels was determined in a tiling box approach, quantifying the fractional vessel volume, *fVV*, with an isotropic resolution of 500 *μ*m on the shrunken tissue. The same cubic subvolumes were used to determine the microvascular density, *MVD*, here defined as the number of individual vessel segments per mm^3^ tissue volume (after shrinkage due to tissue clearing). The vessel length density, ${\rho }_{L}$, and vascular surface density ${\rho }_{A}$, were defined as the total vessel length per shrunken tissue volume in each sample (mm/mm^3^), and as lumen surface area per tissue volume (mm^2^/mm^3^), respectively.

Geometric properties of the individual vessel segments, including mean radius $\bar{r}$, segment length *l* and surface area *A*, were determined using custom Matlab codes on the binary and skeletonized image stacks. A detailed mean radius calculation was implemented along branch lines. The tortuosity $\tau$ of each vessel segment was quantified by the ratio of true vessel length *l* and Euclidean endpoint separation *d* as $\tau =l/d$, often referred to as the distance metric^44,45^.

#### Network topology

To quantify the topology of the vasculature, the branching point connections were modelled as an undirected network by interpreting vessel branching and end points as nodes, interconnected by vessel segments as edges. Utilizing graph theory, the entire systems’ connectivity properties could be quantified, which enables an assessment of topological characteristics on different scales and allows for comparisons with random graph models and other types of complex networks. The topological properties under consideration are described in the following.

#### Scale-free characteristic

In each vascular network, the degree *k* of every node, i.e. the number of attached vessel branches, was determined, delivering the degree distribution *P*(*k*). The relative frequency distributions *P*(*k*) were modelled with a power law: $P(k)\sim {k}^{-\gamma }$, introducing the degree exponent *γ*. It has been found that many real networks exhibit such degree distributions, often with $2\le \gamma \le 3$, identifying them as “scale-free networks^46–48^”.

#### Small-world characteristic

The “small-world” properties^49,50^ can be assessed with three topological measures: the characteristic path length *L*, the network diameter *D*, and the average clustering coefficient *C*. While, along with the total number of nodes *N*, *L* and *D* mirror global network traits, *C* offers insight into the nature of local node connectivity and the tendency towards forming graph theoretical cliques^51^.

The mean clustering coefficient *C* of a network is determined as the average of the clustering coefficients *C*_*i*_ of the individual nodes $i\in \{1,\ldots ,{N}_{n}\}$. The clustering coefficient *C*_*i*_ can be defined as the ratio of the number of edges between the direct topological neighbors of vertex *i* and the maximum number of edges connecting all of its neighbors^51^. For a node *i* with *k*_*i*_ neighbors and *E*_*i*_ connections between these neighbors, the clustering coefficient of the vertex is given by ${C}_{i}=2{E}_{i}/{k}_{i}({k}_{i}-1)$.

The characteristic path length *L*, also called the average shortest path length, describes the mean number of edges on a geodesic to link any two nodes connected by a path on the graph. An implementation of Johnson’s algorithm for the shortest paths problem was used from the MatlabBGL library version 4.0^52^. The small-world property is associated with an exceptionally slow rise in *L* as the network size *N*_*n*_ grows^53^. The network diameter *D* is given by the maximum of all shortest paths, i.e. the greatest node pair separation in the topological sense. The diameter *D* can reflect the degree to which the small-world property is globally persistent.

#### Community unfolding

Expecting a hierarchical branching scheme^2,3,6,7,54^ with a tolerance for locally clustered, lattice-like capillary structures^55–57^, a modularity-based clustering approach was taken in this study. Using the Louvain community unfolding algorithm^58,59^, the networks were partitioned recursively with the aim of maximizing the intracommunity connectivity while keeping intercommunity connections sparse.

The relative dominance of intracommunity edges in a partitioned network can be quantified by the modularity *Q*, defined as^58^:

1 $$Q=\frac{1}{2m}\,\sum _{i,j}\,[{A}_{ij}-\frac{{k}_{i}{k}_{j}}{2m}]\,\delta ({c}_{i},{c}_{j}),$$

with −1 ≤ *Q* ≤ 1. For a weighted network, the adjacency matrix element *A*_*ij*_ holds the weight of the edge connecting nodes *i* and *j*, ${k}_{i}={\sum }_{j}\,{A}_{ij}$ is the weighted degree of node *i*, *c*_*i*_ is the community that node *i* is assigned to, $m=\frac{1}{2}\,{\sum }_{ij}\,{A}_{ij}$ is a normalization factor (the sum over all edge weights) and *δ*(*u*, *v*) is the Kronecker-Delta with $\delta (u,v)=1$ for $u=v$ and $\delta (u,v)=0$ otherwise.

Briefly, the Louvain method starts with every vertex assigned to its own community and then iteratively moves nodes to neighboring communities, always seeking an increase in modularity *Q*. Once a local maximum in *Q* is reached, the algorithm delivers a level of clustering with each node assigned to a community. In the next step, these communities are taken as meta-nodes with the intercommunity connections as edges. The clustering process is repeated recursively on the resulting meta-networks until no more reassignments can increase the modularity and a global maximum in *Q* is reached. Each local maximum in *Q* is expected to reflect the modular structure of the network at a different scale^58^.

#### Community structure

In order to get a more comprehensible view of the giant networks concerning large-scale structures, the spatial distribution and structure of the uncovered communities were studied. We consider a community *j*, comprised of a subset ${Q}_{j}\subseteq \{1,\ldots ,{N}_{n}\}$ of ${n}_{j}=|{Q}_{j}|$ nodes from the total of *N*_*n*_ vertices. With the spatial coordinates ${\overrightarrow{x}}_{q}$, $q\in {Q}_{j}$, of each node assigned to community *j*, the cluster’s node centroid ${\overrightarrow{r}}_{j}$ could be determined: ${\overrightarrow{r}}_{j}={\sum }_{q\in {Q}_{j}}\,{\overrightarrow{x}}_{q}/{n}_{j}$.

The spatial extent *R*_*j*_ of community *j* can be parametrized by the mean Euclidean distance of its constituent nodes $q\in {Q}_{j}$ from the community centroid ${\overrightarrow{r}}_{j}$: ${R}_{j}=\frac{1}{{n}_{j}}\,{\sum }_{q\in {Q}_{j}}\,|{\overrightarrow{x}}_{q}-{\overrightarrow{r}}_{j}|$. In the topological sense, the size of a community is usually determined by its number of nodes *n*_*j*_^53^. In the context of vascular networks, another sensible cluster size parametrization is the number of vessel segments (edges) *e*_*j*_ included in cluster *j*.

A community’s topological perimeter is typically understood as the number of nodes in the community which are involved in connections to other communities^53^. A closely related quantity in the vascular context is the number of connecting edges of cluster *j* to other communities, reflecting the cluster’s supply situation. This measure is regarded as the perimeter *P* in this study.

#### Community connectivity

Each community was treated as a meta-node with weighted intercommunity edges, inherited from connected basic nodes in different clusters. The location of meta-node *j* was interpreted as the community centroid ${\overrightarrow{r}}_{j}$, while its size is reflected by the parameters *n*_*j*_, *e*_*j*_ and *R*_*j*_. The community degree, defined as ${k}_{c,j}=2{e}_{j}+{P}_{j}$, is a measure for the importance of cluster *j* as a supply entity in the network, summarizing size and connectivity in analogy to unclustered networks with allowed self-connectivity.

Graph theoretical quantifications of the clustered meta-networks include the assessment of degree distributions *P*(*k*_*c*_) and degree relations of neighboring communities, clustering coefficients *C*_*c*_, as well as characteristic path lengths *L*_*c*_ and network diameters *D*_*c*_. With the spatial location of every cluster, ${\overrightarrow{r}}_{j}$, the shortest paths between communities were studied depending on their physical separation Δ (${{\rm{\Delta }}}_{ij}=|{\overrightarrow{r}}_{j}-{\overrightarrow{r}}_{i}|$ for communities *i* and *j*).

All statistical testing was conducted using the Kruskal-Wallis-Test, available with Matlab. This is a nonparametric one-way ANOVA, which does not assume a Gaussian distribution of samples.

## Results

### Glioblastoma can mimic the large scale vessel geometry in healthy brain tissue

**Figure 1:**
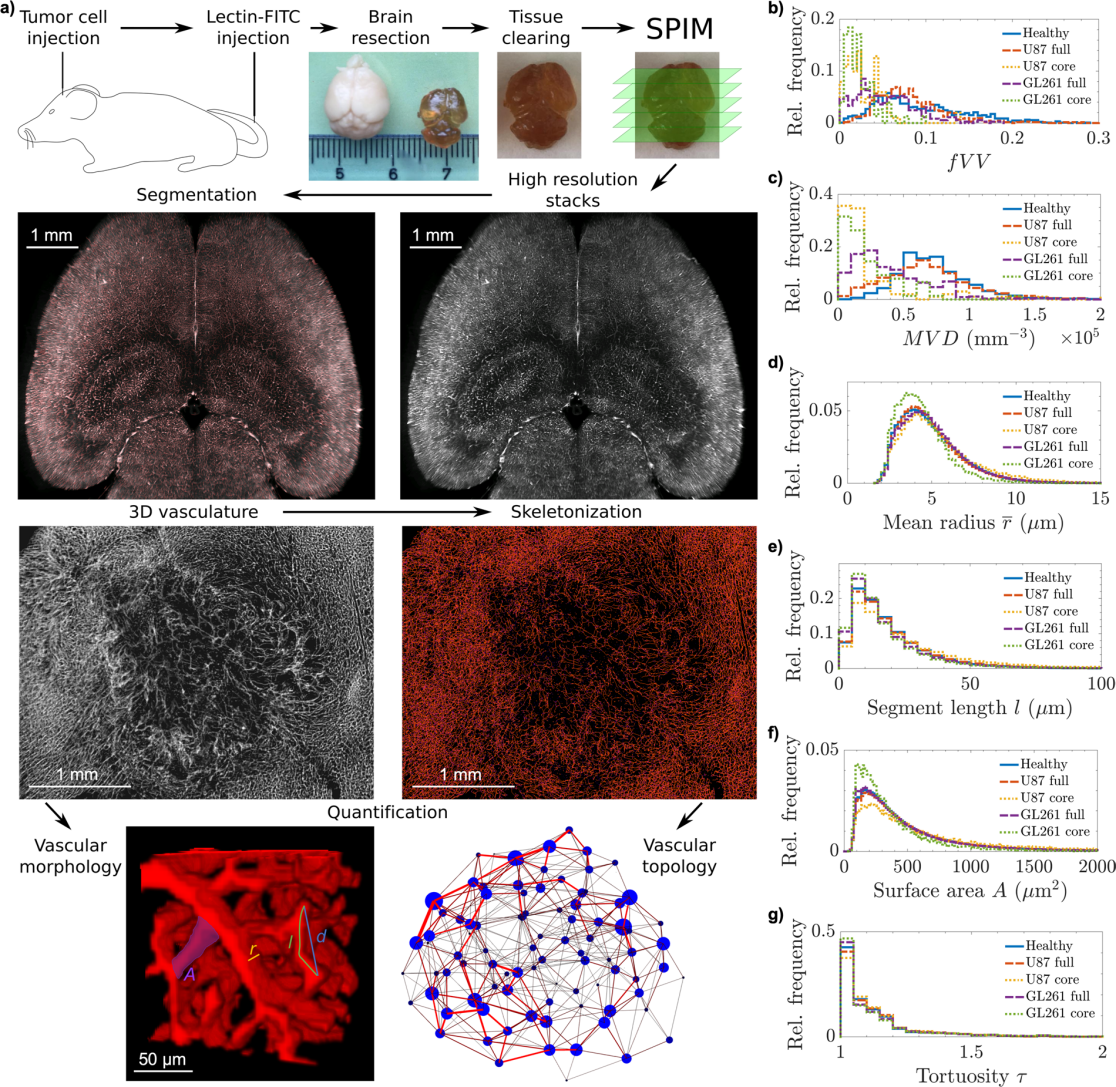
Data acquisition and processing with geometric quantifications. (**a**) Schematic illustration of experimental procedures, including tumor cell and fluorescent marker injections, brain resection and clearing, with photographs of uncleared and cleared brains with cm scale, and Selective Plane Illumination Microscropy (SPIM). In the second row, an original image from a stack of a healthy mouse brain is presented on the right, with the binary segmentation overlay in red to the left (see Supplementary Movies 1, 2 and 3 for segmentation results in more detail). Below the brain segmentation image, an average intensity projection from a 200 *μ*m thick section of a segmented, noise-filtered, and hole-filled image stack of a U87 glioblastoma is shown. To the right, the skeletonized version of the same dataset is presented, with branch voxels in orange and branching points in magenta. The vascular network quantifications on this post-processed data are illustrated in the last row. The vascular morphology assessment is clarified in a cube of 130 *μ*m side length, marking a radius value *r*, length *l* and endpoint-separation *d*, as well as a segment’s surface area *A*. Using the vascular skeleton, the network topology is studied, which is illustrated by a clustered graph, presenting the spatial distribution of vessel communities in a U87 glioblastoma. From the geometric quantifications, relative frequency distributions of (**b**) fractional vessel volume *fVV* and (**c**) microvascular density *MVD* in cubes with 500 *μ*m side length, and distributions of geometric characteristics of all individual vessel segments are presented: (**d**) mean vessel radius $\bar{r}$, (**e**) segment length *l*, (**f**) surface area *A*, and (**g**) segment tortuosity $\tau$.

The data acquisition process for the results presented is illustrated in Fig. 1a. In healthy controls, we found a mean fractional vessel volume of $\langle \,fV{V}_{h}\rangle =9.8\pm 3.3 \%$ (with standard error of mean), which is in reasonable agreement with documented values of an intracranial mean $\langle \,fV{V}_{ic}\rangle =5.8\pm 0.4 \%$ and maximum $\langle \,fV{V}_{{\max }}\rangle \approx 7.9 \%$ in the medulla and cerebral cortex, determined from micro-CT measurements at 20 *μ*m isotropic resolution^60^. The incorporated 3D volume tiling with 500 *μ*m cubes comprised a total of 1265 boxes in healthy tissue, 871 in U87 tumors (101 in the core), and 364 in GL261 tumors (76 in the core). In total, approximately 4.4 million healthy vessel segments were compared to 1.8 million vessels in U87 glioblastoma (21 400 in the core region) and 380 000 vessels in GL261 tumor tissue (22 200 in the core). Table 1 summarizes the mean geometric properties of the tissue samples under consideration.

**Table 1:** Global tissue properties from *n* = 6 healthy brain hemispheres (healthy networks) and tumor specimens (full networks and exclusively tumor cores).

	〈*V*〉 mm^3^	〈*fVV*〉%	〈*MVD*〉 · 10^3^ mm^−3^	〈${{\boldsymbol{\rho }}}_{{\boldsymbol{L}}}$〉 mm^−2^	〈${{\boldsymbol{\rho }}}_{{\boldsymbol{A}}}$〉 mm^−1^	〈$\bar{{\boldsymbol{R}}}$〉 *μ*m	〈*l*〉 *μ*m	〈*A*〉 *μ*m^2^	$\tilde{{\boldsymbol{\tau }}}$	${{\boldsymbol{\tau }}}_{{\bf{95}}}$
Healthy networks	14.7 ± 4.1	10.3 ± 3.7	53 ± 10	980 ± 99	25.4 ± 10.0	$$4.9\begin{array}{c}+1.7\\ -1.2\end{array}$$	$$19\begin{array}{c}+15\\ -9\end{array}$$	$$505\begin{array}{c}+472\\ -240\end{array}$$	1.071	1.453
U87 full networks	8.3 ± 4.3	7.2 ± 1.6	38 ± 12	734 ± 145	16.8 ± 4.4	$$4.9\begin{array}{c}+1.7\\ -1.2\end{array}$$	$$20\begin{array}{c}+16\\ -10\end{array}$$	$$547\begin{array}{c}+566\\ -267\end{array}$$	1.079	1.463
U87 core networks	0.3 ± 0.3	3.0 ± 1.4	11 ± 8	268 ± 146	6.1 ± 3.9	$$5.4\begin{array}{c}+2.2\\ -1.5\end{array}$$	$$25\begin{array}{c}+24\\ -13\end{array}$$	$$806\begin{array}{c}+1089\\ -446\end{array}$$	1.080	1.481
GL261 full networks	2.8 ± 1.0	4.8 ± 0.9	23 ± 7	413 ± 74	13.8 ± 8.4	$$5.0\begin{array}{c}+1.7\\ -1.3\end{array}$$	$$18\begin{array}{c}+16\\ -9\end{array}$$	$$491\begin{array}{c}+466\\ -235\end{array}$$	1.070	1.553
GL261 core networks	0.3 ± 0.2	1.9 ± 0.1	13 ± 9	214 ± 103	7.5 ± 5.7	$$4.4\begin{array}{c}+1.3\\ -1.0\end{array}$$	$$17\begin{array}{c}+16\\ -8\end{array}$$	$$386\begin{array}{c}+361\\ -177\end{array}$$	1.066	1.557

Means with standard deviation (SD) are given for the tissue volume of each specimen *V* (after shrinkage from clearing), fractional vessel volume *fVV*, microvascular density *MVD*, total vessel length density ${\rho }_{L}$ (in mm/mm^3^), and vascular surface density ${\rho }_{A}$ (in mm^2^/mm^3^). Arithmetic means and average directed deviations of geometric vessel properties with log-normal distributions, namely mean radius $\bar{r}$, segment length *l* and surface area *A*. The exponentially distributed segment tortuosity $\tau$ is characterized by the median $\tilde{\tau }$ and 95%-quantile ${\tau }_{95}$.

Distributions of the fractional vessel volume *fVV* and microvascular density *MVD*, determined over 500 *μ*m cubes, were positively skewed in all tissue types (Fig. 1b,c). Values were significantly lower in full GL261 tumors (*fVV*: *p* = 0.007; *MVD*: *p* = 0.004 from testing with 6 vs. 6 sample means), while U87 glioblastoma showed density distributions similar to the healthy controls, when including the periphery (Table 1). The U87 tumor cores exhibited more heterogeneity than the GL261 models. On the 500 *μ*m scale, the U87 tumors featured regions with considerably decreased branching density *MVD*, while the *fVV* did not show matching voids (Fig. 1b,c). While vessel calibres and lengths were virtually unchanged in the full U87 specimens, the core vessels showed considerably larger radii and branching lengths, which can account for heightened *fVV* at relatively low *MVD*. The GL261 model showed opposite trends in the core, with shorter, thinner vessels, resulting in lower *fVV* despite higher *MVD*. The vessel length density ${\rho }_{L}$ was significantly lower in all tumor samples compared with healthy controls (U87 full: $p=0.01$; GL261 and cores: $p=0.004$ from testing with, respectively, 6 sample means, Table 1).

The increased vessel length in U87 glioblastoma, and especially its core, suggests suppressed branching and vessel occlusion, while the shift towards shorter segment lengths and smaller radii suggest more active angiogenesis in the GL261 tumor core (Fig. 1d–f). The more sharply peaked radius distribution suggests a flattened branching hierarchy in glioblastoma with respect to healthy vasculature^3^. The lumen surface area *A* per vessel segment is correlated with the length *l* and radius $\bar{r}$, showing more clearly the opposing trends in U87 and GL261 vessel remodeling. The vessel tortuosity $\tau$ approximately followed shifted exponential distributions (with $\tau \ge 1$ by definition) in all tissue types (Fig. 1g) and is characterized by the median $\tilde{\tau }$ and 95%-quantile ${\tau }_{95}$ in Table 1.

The vessel tortuosity $\tau$ again showed distinct alterations in each tumor model. In U87 glioblastoma and its core, both the median $\tilde{\tau }$ and upper quantile ${\tau }_{95}$ increased moderately from healthy controls to tumor cores ($p < 0.01$ for core vs. control). In contrast, in GL261 models, the median $\tilde{\tau }$ decreased towards the core, while ${\tau }_{95}$ strongly increased ($p < 0.01$ for full networks and cores vs. controls); the GL261 tumors upheld a large number of relatively straight segments with several very tortuous ones. Such heterogeneity with a tendency towards increased tortuosity is consistent with the findings of previous studies^37^. Analogous tests against healthy tissue on the remaining geometric vessel properties did not indicate statistical significance ($\langle \bar{r}\rangle$: $p > 0.4$; $\langle l\rangle$: $p > 0.1$; $\langle A\rangle$: $p > 0.1$ for all tumor sets, including cores). Extended sample sizes could show that the mean vessel tortuosity, if accessible, may serve as a biomarker for tumor vasculature, supporting previous findings in humans^61,62^.

### Altered network topology in glioblastoma multiforme

We present the first topological quantifications on cerebrovascular networks of such size and resolution. The custom-written codes were validated on functional human brain networks previously quantified^63^ to assure correct numerical implementations.

#### Heterogeneous effects on local vessel connectivity in different glioblastoma types

By modeling branching and vessel end points as the nodes of a network, interconnected by vessel segments as edges, the connectivity in such large systems can be characterized using graph theory^41,64^. In accordance with an elevated *MVD* (edge density), the larger healthy tissue samples also featured higher node densities ${\rho }_{n}$, leading to healthy networks of larger size *N*_*n*_ and *N*_*e*_ compared with tumor networks (Table 2).

**Table 2:** Mean basic network properties with SD among, *n* = 6 healthy and tumor-bearing specimens, respectively.

	〈${{\boldsymbol{\rho }}}_{{\boldsymbol{n}}}$〉 · 10^3^ mm^−3^	〈*N*_*n*_〉 · 10^3^	〈*N*_*e*_〉 · 10^3^	〈$\bar{{\boldsymbol{k}}}$〉	〈*k*_*max*_〉	〈*γ*〉	〈*C*〉	〈*β*〉
Healthy networks	35.7 ± 13.7	505 ± 207	817 ± 330	3.24 ± 0.11	20.7 ± 4.1	8.72 ± 1.18	0.049 ± 0.012	2.36 ± 0.01
U87 full networks	27.4 ± 13.0	196 ± 99	286 ± 150	2.89 ± 0.15	17.3 ± 3.4	8.53 ± 1.38	0.056 ± 0.010	2.36 ± 0.01
U87 core networks	9.3 ± 6.4	3 ± 3	4 ± 4	2.29 ± 0.26	8.5 ± 2.6	2.79 ± 4.52	0.078 ± 0.021	2.2 ± 0.4
GL261 full networks	19.1 ± 5.1	52 ± 21	80 ± 36	3.06 ± 0.16	31.5 ± 9.2	5.35 ± 0.87	0.123 ± 0.019	1.4 ± 0.2
GL261 core networks	10.5 ± 7.0	3 ± 3	5 ± 5	2.67 ± 0.36	15.7 ± 8.6	4.18 ± 2.20	0.144 ± 0.004	1.6 ± 0.3
Random networks	31.6 ± 13.4	358 ± 216	561 ± 359	3.22 ± 0.19	14.3 ± 0.9	n.${\rm{a}}$.	(1 ± 1) · 10^−5^	n.${\rm{a}}$.

Node density in (shrunken) tissue volume ${\rho }_{n}$, as well as the total number of branching nodes *N*_*n*_ and edges *N*_*e*_ per specimen. Mean local connectivity measures from all healthy and tumor specimens, including mean node degree $\bar{k}$, maximum degree per specimen *k*_*max*_, and clustering coefficient *C*; the scaling exponent *β* from fitting ${C}_{i}({k}_{i})\sim {k}_{i}^{-\beta }$ is given with SD among samples. For comparison, corresponding quantities are also given for *n*_*r*_ = 12 Erdös-Rényi graphs^66^ with node and edge numbers equal to the healthy and full U87 networks. n.a.: not applicable.

**Figure 2:**
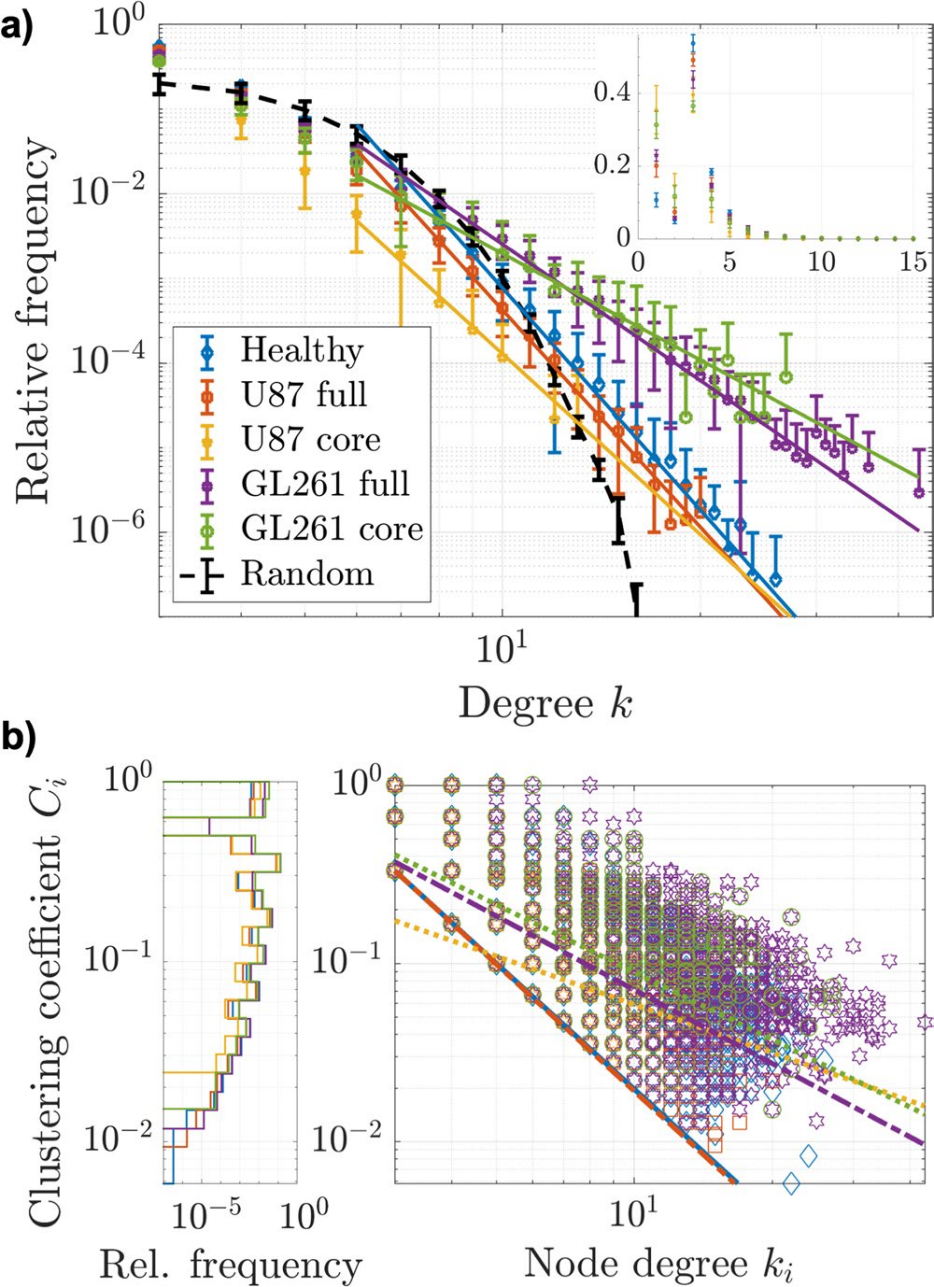
Vascular network topology. (**a**) Degree distributions (mean with SD among samples) from *n* = 6 healthy brain hemispheres, full U87 and GL261 tumors, and tumor cores, respectively. For comparison, the mean degree distribution from ${n}_{r}=12$ random Erdös-Rényi graphs^66^ with the corresponding node and edge numbers, is displayed as well, following a Poisson distribution. The large plot presents the distributions for $k\ge 3$ on logarithmic axes, while the inlayed plot shows the full distributions on linear scales. The logarithmic plot includes straight lines in corresponding colors, representing power law fits to the vascular data. (**b**) Bivariate distributions of node clustering coefficients *C*_*i*_ with corresponding node degrees *k*_*i*_, including all nodes with $k\ge 3$ from all datasets and power law fits in corresponding colors. The marginal distributions of clustering coefficients *C*_*i*_ are displayed with a logarithmic ordinate axis to better illustrate differences along the entire range.

The degree *k* of a node corresponds to the number of vessels meeting at that vertex. The vascular skeletons exhibited high degree nodes, presumably at the intersection between arterial and venous tree branches with the dense capillary mesh^5,20^ or in angiogenic hotspots^65^, especially in the tumor periphery. In Supplementary Movies 4 and 5, we show vascular nodes with degree *k* = 24 from a healthy network, and *k* = 14 from the U87 tumor periphery. The healthy microvascular networks consistently featured higher degree vertices than the U87 vessel networks, while the GL261 periphery yielded the highest *k*_*max*_ (Table 2 and Fig. 2a). The increased abundance of terminal branches with degree *k* = 1 in both tumor models is a strong indicator for angiogenesis (see inlay in Fig. 2a). Vessel endpoints constituted 20 ± 3% of all nodes in full U87 networks (36 ± 7% in the core) and 23 ± 2% of nodes in the GL261 networks (32 ± 4% in core), in contrast to 11 ± 2% in healthy networks (with SD among samples). In consequence, the mean node degree $\langle \bar{k}\rangle$ was decreased in both tumor models (Table 2). Nodes with degree $k=2$ are an artefact of skeletonization and dealt with in the discussion.

In Table 2, mean local connectivity measures are given for the vascular networks, as well as comparable random graphs^66^. Since the random networks were constructed from the same number of constituents as the healthy and U87 tumor networks, the node density ${\rho }_{n}$, the size parameters *N*_*n*_ and *N*_*e*_, and the mean node degree $\langle \bar{k}\rangle$ are identical to the weighted means over the reference networks. Yet, as expected, the vascular networks are subject to greater organization and heterogeneity, which reflects in the considerably higher maximum degree *k*_*max*_ at unchanged mean degree $\bar{k}$.

The degree distributions of the vascular networks approximately obey a power law $P(k)\sim {k}^{-\gamma }$ for higher degrees, with least squares fits for $k\ge 5$ showing good approximations of the vascular data (Fig. 2a). Despite unusually large exponents, this classifies the healthy and pathological vasculature as scale-free networks^53^, placing them in line with many complex networks in nature, including the human brain^63^, metabolic^67^, and protein networks^68^. While both tumor models decreased the exponent *γ* with respect to healthy networks, the change was much stronger in GL261 tumors when comparing full networks, whereas U87 cores showed the highest heterogeneity, followed by GL261 cores (see mean $\langle \gamma \rangle$ in Table 2).

The clustering coefficient *C* quantifies the degree to which a node’s neighboring nodes are well interconnected. Real, scale-free networks often have much higher clustering coefficients than comparable random networks, even with quasi-identical degree distributions^53,69^. Generally, the studied vascular networks presented much higher clustering than corresponding random graphs with mean $\langle {C}_{r}\rangle =(1\pm 1)\cdot {10}^{-5}\approx \langle \bar{k}/{N}_{e}\rangle$^53^. The tumor vasculature exhibited an increased mean clustering coefficient $\langle C\rangle$ compared to healthy networks, which, by definition, indicates a higher abundance of local vessel loops. Both pathological models showed stronger clustering in the tumor core, but the effects were much more amplified in GL261 tumors (Table 2).

Distributions of individual node clustering coefficients *C*_*i*_ with corresponding degrees *k*_*i*_ are presented in Fig. 2b. U87 tumor networks had a slightly higher relative number of cliques around low degree branching points ($k\le 5$), increasing the abundance of ${C}_{i}=1$ nodes. Although in rare occurrence, the healthy vessel networks featured nodes with slightly elevated clustering for most degree values above $k=5$, as compared to the U87 tumors, while GL261 tumors showed significantly increased clustering, also for high degree nodes ($p < 0.004$ for GL261 cores and full tumors; $p=0.025$ for U87 cores and $p > 0.1$ for full U87 networks compared to controls, tested with mean *C* per tissue specimen).

The approximate scaling of node clustering coefficients with ${C}_{i}({k}_{i})\sim {k}_{i}^{-\beta }$ has been identified as a hallmark of networks with hierarchical structure^70^. Robust power-law fits on the individual node values yielded the scaling exponents *β* given in Table 2. Despite the large spread of clustering coefficients *C*_*i*_ (Fig. 2b), the healthy and pathological networks studied here can be classified as hierarchical networks.

#### Reshaped nonlocal connectivity

**Figure 3:**
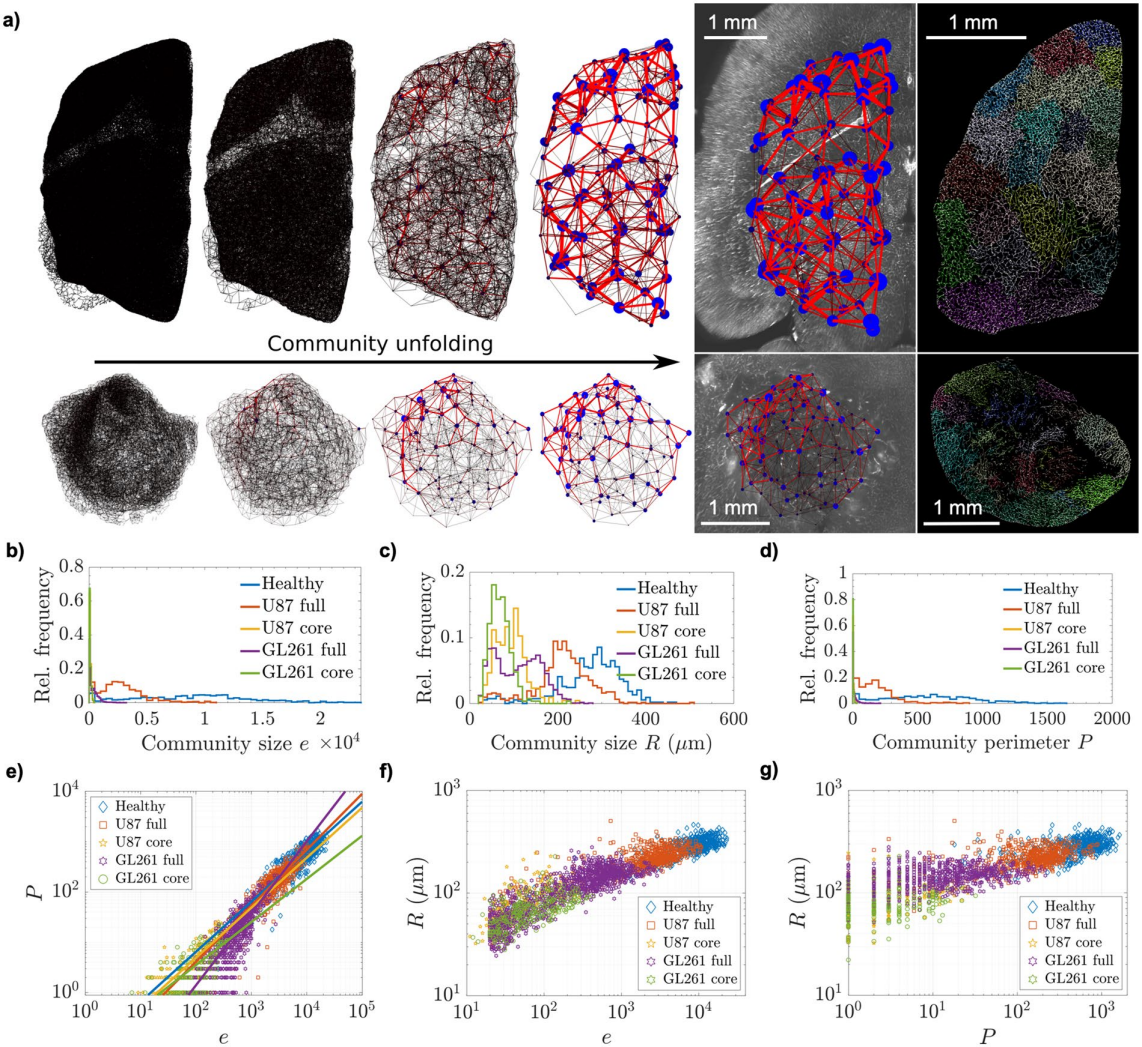
Modular network structure. (**a**) Schematic graphs of the community unfolding process on an entire vascular network in a healthy brain hemisphere (top) and full U87 glioblastoma (bottom). Each level of partitioning represents a local maximum in modularity *Q*, attained with increasing community sizes. The rightmost graph shows the clustering scheme with global maximum modularity over a central slice of the original SPIM-image. Communities are depicted by circles with diameter and brightness (blue) proportional to cluster size *e*_*j*_, while the weight of a connection (the number of intercommunity vessel segments) is encoded in the edge thickness and brightness (red). Cluster positions are given by their centroid ${\overrightarrow{r}}_{j}$. The specimens encompass comparable (shrunken) tissue volumes of ${V}_{h}=12.11\,{{\rm{mm}}}^{3}$ and ${V}_{g}=12.87\,{{\rm{mm}}}^{3}$ in healthy control and tumor tissue, respectively (excluding ventricular space in the healthy brain, blinded for analysis). To the right of the partitioning chains, projections of 100 *μ*m thick sections of the skeletonized vessel data show community affiliation (at global maximum *Q*) through the color of each branch segment. Relative distributions of community size properties from all specimens follow, namely (**b**) internal number of edges *e*, (**c**) mean physical extent *R*, and (**d**) community perimeter *P*. Panel (e) presents the relationship between a community’s number of internal edges *e* and its perimeter *P*. Linear fits to the log-log-representation are plotted in lighter colors over the datapoints, presenting slopes $\xi$. The following plots illustrate the relationships between (**f**) community edges *e* and mean physical extent *R*, as well as (**g**) perimeter *P* and *R*.

In order to quantify the vascular networks’ nonlocal topology, the branching nodes were clustered based on modularity, using the Louvain community unfolding algorithm^58^. An exemplary consecutive community unfolding process on a full U87 glioblastoma and healthy brain hemisphere is presented graphically in Fig. 3a. For the topological quantifications reported in the following, the partitioning schemes corresponding to global maximum modularity *Q* were used for each network (in Fig. 3a, the rightmost community networks). To suppress boundary effects, isolated communities (disconnected vessel clusters) including less than twenty edges were removed from our analysis.

#### Tumor-induced decomposition of large-scale community structures

The Louvain algorithm unveiled considerably larger clusters in healthy vessel networks than it did in glioblastoma vasculature, with dramatic differences in tumor cores and amplified effects in the GL261 model. This reflects in the communities’ number of nodes *n*, number of vessel edges *e*, and the mean physical extent *R*, as well as the community perimeter *P*, i.e. the number of vessels to neighboring communities, with broader distributions in healthy networks and much smaller modular communities in all tumors (Table 3 and Fig. 3b–d).

**Table 3:** Structural properties of communities uncovered in the vascular networks.

	〈*Q*〉	〈*N*_*c*_〉	〈*N*_*ice*_〉 · 10^3^	〈${{\boldsymbol{\rho }}}_{{\boldsymbol{c}}}$〉 mm^−3^	〈${{\boldsymbol{\rho }}}_{{\boldsymbol{ice}}}$〉 · 10^3^ mm^−3^	〈$\bar{{\boldsymbol{n}}}$〉 · 10^3^	〈$\bar{{\boldsymbol{e}}}$〉 · 10^3^	〈$\bar{{\boldsymbol{R}}}$〉 *μ*m	〈$\bar{{\boldsymbol{P}}}$〉
Healthy networks	0.51 ± 0.01	88 ± 21	25.18 ± 9.35	6 ± 2	1.8 ± 0.8	5.6 ± 3.1	8.8 ± 5.1	275 ± 74	573 ± 344
U87 full networks	0.50 ± 0.02	112 ± 30	8.17 ± 4.35	14 ± 7	1.2 ± 0.7	1.9 ± 1.3	2.8 ± 2.1	211 ± 65	173 ± 143
U87 core networks	0.43 ± 0.06	36 ± 24	0.08 ± 0.12	179 ± 147	0.3 ± 0.4	0.07 ± 0.06	0.09 ± 0.08	94 ± 35	5 ± 6
GL261 full networks	0.57 ± 0.03	204 ± 50	1.38 ± 0.89	77 ± 18	0.5 ± 0.3	0.3 ± 0.3	0.4 ± 0.5	110 ± 51	14 ± 26
GL261 core networks	0.48 ± 0.12	45 ± 30	0.06 ± 0.08	176 ± 58	0.2 ± 0.3	0.06 ± 0.05	0.09 ± 0.08	70 ± 25	3 ± 5

Mean values with SD among samples are given for the final partitioning modularity *Q*, the number of communities per specimen *N*_*c*_, the number of intercommunity edges *N*_*ice*_, the mean number of communities and intercommunity edges per mm^3^ (shrunken) tissue volume, ${\rho }_{c}$ and ${\rho }_{ice}$, respectively, the mean number of nodes $\bar{n}$ and edges $\bar{e}$ per community, as well as mean physical extent $\bar{R}$ and perimeter $\bar{P}$ of the communities within each sample.

At the highest partitioning level, determined by Eq. (1), the mean maximum modularity in healthy control networks was approximately 0.5. This modularity was maintained in tumors, but with much smaller vessel clusters (see modularities $\langle Q\rangle$ and node numbers $\langle \bar{n}\rangle$ in Table 3). While the modularity was slightly lower in tumor cores, it was even increased in GL261 tumors, compared to healthy controls, following a drastic breakdown of large vessel communities. Corresponding Erdös-Rényi networks, clustered with the same procedure, yielded maximum modularity close to zero, with mean value ${\hat{Q}}_{r}=(6\pm 32)\cdot {10}^{-7}$ from ${n}_{r}=12$ random networks. Practically, the same modularity was maintained in healthy and pathological vessel networks, but on substantially different community size scales.

The clustering sequence in Fig. 3a demonstrates that the healthy brain exhibits a more uniform distribution of differently sized clusters throughout the tissue, while the glioblastoma upholds large vessel communities asymmetrically at its boundaries. The rightmost images show that modular clusters are disrupted and separated in the glioblastoma, and vessel communities are not as dense or large as they are in the healthy brain. This indicates that tumor-induced vessel remodeling leads to a breakdown of pre-existing topological clusters in order to form smaller supply entities, which could be regulated more independently.

The correlation between community size *e* and perimeter *P* can be associated with the isolation of modular vessel communities. Robust power-law fits to the roughly linear relationship on logarithmic axes (Fig. 3e), assuming $P(e)\sim {e}^{\xi }$, yielded the exponents $\xi$ in Table 4 (corresponding to the slopes plotted in Fig. 3e). An analogous quantification on large parts of the cortical vasculature in a mouse model documented an exponent of $0.83\pm 0.04$^30^, where values between 2/3 and 1 were interpreted as a manifestation of weak community structure, while lower scaling exponents $\xi$ would indicate the persistence of strongly isolated communities. Our results show that community interconnectivity differs in tumor core and periphery, but both tumor models showed consistent changes from healthy vasculature. Full tumor networks showed an increased exponent $\xi$ with relatively little deviation from the assumed relationship. In contrast, tumor cores had lowered exponents with large uncertainty and stronger variance, with more pronounced differences to healthy tissue in the GL261 models.

**Table 4:** Connectivity between communities.

	*ξ*	〈*e*/*P*〉	〈${\bar{{\boldsymbol{k}}}}_{{\boldsymbol{c}}}$〉	*κ*	〈${\bar{{\boldsymbol{k}}}}_{{\boldsymbol{c}},{\boldsymbol{u}}}$〉	〈*C*_*c*_〉	〈*L*_*c*_〉	〈*D*_*c*_〉
Healthy networks	0.99 ± 0.01	18 ± 11	17780 ± 5930	0.18 ± 0.01	8.8 ± 1.7	0.51 ± 0.02	3.1 ± 0.4	7.3 ± 1.1
U87 full networks	1.11 ± 0.01	22 ± 35	5360 ± 2760	0.37 ± 0.01	9.2 ± 0.9	0.50 ± 0.04	3.1 ± 0.4	7.5 ± 1.4
U87 core networks	0.99 ± 0.12	27 ± 23	140 ± 130	0.88 ± 0.03	2.3 ± 1.1	0.28 ± 0.22	2.6 ± 1.0	5.7 ± 2.2
GL261 full networks	1.43 ± 0.01	58 ± 71	780 ± 370	0.87 ± 0.02	5.5 ± 0.9	0.40 ± 0.05	4.4 ± 0.4	11.0 ± 1.8
GL261 core networks	0.86 ± 0.15	39 ± 33	140 ± 100	0.87 ± 0.03	2.0 ± 0.8	0.17 ± 0.21	2.4 ± 1.3	6.0 ± 4.2

Isolation scaling exponents *ξ* from robust fits assuming $P(e)\sim {e}^{\xi }$, mean number of internal vessel segments per intercommunity edge *e*/*P*, mean community degree ${k}_{c}=2e+P$ and assortativity exponent *κ* from fits approximating the neighboring degree relationship with $\langle {k}_{c1}\rangle \,({k}_{c})\sim {k}_{c}^{\kappa }$, mean number of unique topological neighbor-communities ${\bar{k}}_{c,u}$, community clustering coefficient *C*_*c*_, characteristic path length *L*_*c*_ and diameter *D*_*c*_ of the meta-networks, averaged from all specimens, and given with SD.

For each vessel segment connecting a cluster to another, on average, a vessel community incorporated $\langle e/P\rangle$ internal edges (sample mean with SD from all communities given in Table 4). All types of vascular networks exhibited pronounced modular structures. Tumor vasculature exhibited higher heterogeneity in community isolation with a tendency towards reduced cluster connectivity (Fig. 3e). Whereas communities were most modular in full GL261 networks including the periphery, the U87 model showed stronger community isolation in its core, with higher heterogeneity in tumor peripheries for both models.

Furthermore, the vessel communities in tumor tissue presented higher heterogeneity in vessel segment densities, especially towards lower values (Fig. 3f; tumor networks featured communities with relatively large spatial extent *R* and low edge number *e*). The glioblastoma upheld vessel clusters with a low number of intercommunity segments *P* in a wide range of edge numbers *e* (Fig. 3e) and mean cluster radii *R* (Fig. 3g). The tumor networks not only presented a significant breakdown of vascular community size, but also decomposed connectivity among existing communities.

The identified vessel communities form meta-networks on larger length-scales of several hundred micrometers. The connections between communities can be interpreted as weighted edges between community nodes (see graph illustration in Fig. 3a). Standard graph theoretical measures, derived from the undirected meta-networks of healthy and pathological vascular communities, revealed profound characteristics in the organization of tumor-specific vessel clusters. Effects were again more pronounced in GL261 tumors, but from both models studied here, general trends may be extracted from our results.

**Figure 4:**
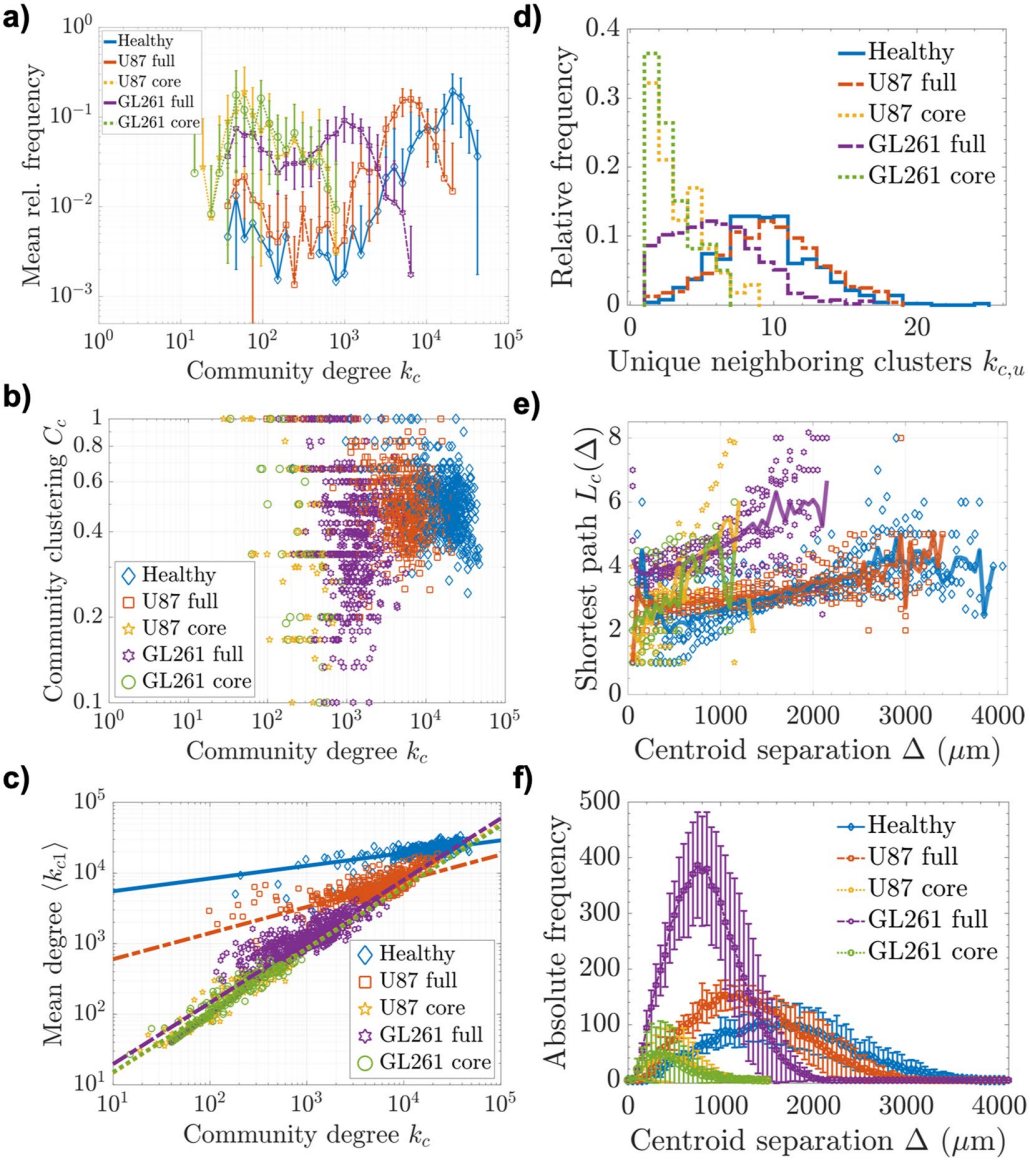
Community interconnectivity. (**a**) Mean log-binned frequency distributions of community degree ${k}_{c}=2e+P$ (with SD among samples), (**b**) community clustering coefficients *C*_*c*_ vs. *k*_*c*_, and (**c**) mean degree of neighboring communities $\langle {k}_{c1}\rangle$ vs. *k*_*c*_ with fits $\langle {k}_{c1}\rangle \,({k}_{c})\sim {k}_{c}^{\kappa }$. (**d**) Relative frequency distributions of the number of unique topological neighbor communities *k*_*c*,*u*_, (**e**) separation-dependent shortest path length *L*_*c*_ between two connected communities, separated by the Euclidean distance Δ ± *δ*/2 with increments of *δ* = 50 *μ*m. The datapoints represent individual community-pair instances and the brighter lines connect the mean values over all datasets for each distance bin in Δ. (**f**) The number of community pairs with centroid separation Δ ± *δ*/2 (with SD among samples).

The importance of a community as a supply entity is reflected by the community degree ${k}_{c}=2e+P$, adapting the classical notion of the degree of a node with *P* connections to other nodes and *e* connections to itself (internal vessel edges). The clustered meta-networks did not show scale-free properties and degree distributions mainly reflected the breakdown of large vessel communities in healthy tissue. This effect was more pronounced in full GL261 networks, but produced similar degree distributions in the cores of both tumor models (Table 4 and Fig. 4a). In contrast to the basic vessel networks analyzed before, the meta-networks presented reduced clustering coefficients *C*_*c*_ between communities in glioblastoma, with higher heterogeneity and lower interconnectivity in tumor cores (Table 4 and Fig. 4b). The community clustering coefficients *C*_*c*_ did not present a distinct dependence on the degree *k*_*c*_, advocating a loss of the basic networks’ hierarchical organization in the large-scale meta-networks (Fig. 4b).

We observed a positive correlation between the degrees of directly connected communities in all vascular networks (Fig. 4c), which indicates that vessel communities are subject to assortative mixing^71,72^. Such mixing is based on large, well-connected communities that are preferentially attached to other communities of similar importance; an unexpected finding, as spatial networks typically yield flat $\langle {k}_{c1}\rangle$ distributions^73^. As robust power law fits assuming $\langle {k}_{c1}\rangle \,({k}_{c})\sim {k}_{c}^{\kappa }$ emphasized, the assortativity was more pronounced in glioblastoma vasculature (exponents $\kappa$ in Table 4 shown as slopes of straight lines in Fig. 4c). Both tumor models presented very similar community sorting in the core, with little difference in entire GL261 networks, but more similarity of full U87 networks with healthy cerebrovasculature. A significant increase of assortative mixing in tumor vasculature is clear in both models.

The communities in GL261 tumor tissue and all tumor cores tended to be connected to a smaller number of distinct neighboring clusters, while in the full U87 networks, communities had slightly more topological neighbors than healthy networks. This indicates abnormally high community interconnectivity in the U87 periphery (Fig. 4d). A comparison of the mean number of unique neighboring clusters $\langle {\bar{k}}_{c,u}\rangle$ (Table 4) with the mean community perimeter $\langle \bar{P}\rangle$ (Table 3) shows that intercommunity connections in a healthy network are often enforced by many more individual vessel segments. With much larger healthy communities (Table 3), this observation makes sense when considering the supply and drainage functions of the intercommunity connections to the clusters, and it supports the notion of reduced community interconnectivity in tumor tissue.

In nonlocal connectivity, U87 and GL261 tumor-derived community networks presented diverse properties. Considerably higher densities *ρ*_*c*_ of much smaller vessel communities in tumor tissue can be associated with a rise in mean topological diameter *D*_*c*_, i.e. the longest path through the network over communities, and mean path length *L*_*c*_ between any pair of connected communities, in relativity to reduced tumor network sizes in comparison to healthy controls (Table 4). Nevertheless, the vascular networks in tumor tissue exhibited relatively small topological path length increases, which becomes more apparent in dependence of the physical separation Δ between the community centroids (Fig. 4e; the number of occurrences for each pair separation bin is presented in Fig. 4f).

The mean path length *L*_*c*_(Δ) in full U87 tumor networks was not appreciably higher than in healthy tissue, even for large distances between community centroids in the millimeter range (Fig. 4e). U87 tumor cores and GL261 specimens were subject to more heterogeneity, with mean path lengths exhibiting a steeper rise with physical separation, but also over considerably smaller communities (see *n*, *e*, *R* in Table 3). Our results show that the well-connected U87 tumor periphery facilitates short path lengths between virtually all communities in the tumor. Despite a decreased intercommunity edge density ${\rho }_{ice}$ and communities with considerably smaller physical extent *R* and vessel numbers *n* (Table 3 and Fig. 3b,c), tumors maintained relatively short topological separations between communitites, even over large distances through the tissue.

## Discussion

We characterized entire, perfused vascular systems in healthy mice brain, U87- and GL261-glioblastoma xenografts using basic geometric and network theoretical measures. The U87 cell line is known to have undergone genetic drift over recent years, growing in a solid, bulky manner instead of promoting diffuse infiltration of the brain parenchyma, like most human gliomas^74^. Furthermore, this tumor model has been found to have anomalous microvascular properties, with vessel distributions rather resembling healthy vasculature than other tumors in some aspects^75^. The solid growth pattern of the U87-glioma aided in delineating the tumor tissue from healthy tissue to attain first quantifications of entire tumor-immanent vascular networks, with the highly angiogenic GL261 model serving for further comparison. The methods presented here can be applied to arbitrary 3D-image data, independent of the imaging technique, and therefore, they are transferable to humans and other pathologies.

Our results render the tumor tissue to be irregularly perfused with a high variation in local vessel densities and characteristic differences in core and periphery, consistent with general knowledge^76,77^. We did not observe any significant increase in blood volume fraction *fVV* or mean vessel radii $\bar{r}$, as has been documented with different experimental methods and brain tumors, including the U87 cell line^78–82^. This is expected to be attributed to the tumor stage examined and the collective character of the geometric comparisons, which stand in contrast to local, selective analyses of angiogenic regions^83^.

In our integrative study of the full vascular network, we found U87 tumor tissue to feature very low vessel densities *MVD*, but relatively unchanged blood volume fractions *fVV* in some regions on the 500 *μ*m scale. Increased vessel calibre in the U87 tumor core indicates hypoxic vasodilation, which can compensate the tissue’s fractional blood volume with very little perfused vessels^84^. While the main hallmarks of tumor angiogenesis are believed to be an elevated vessel density *MVD* and fractional blood volume *fVV*, dilated vessel radii $\bar{r}$, higher tortuosity $\tau$, and decreased branching lengths *l*^85^, our comparisons with vasculature from different regions of the healthy brain did not reveal elevated vessel densities significant on a global scale. The *MVD* and *fVV* were overall decreased in glioblastoma, while branching lengths *l* and vessel radii $\bar{r}$ changed distinctly in both tumor models. Previous studies have shown that the above-mentioned properties, relating to vascular density, can change and decrease with tumor progression^83^, shifting angiogenic activity to the tumor periphery, while reducing the perfusion density in the core^77,86^.

Our quantifications of healthy cerebrovascular networks included many brain regions with heterogeneous perfusion densities, without a differentiation of vessel types. The healthy networks featured high calibre arteries and veins that increased the average vessel radius. The tumor networks were in deficit of such large vessels, but presented a shift of small capillaries towards higher calibres, with the exception of GL261 tumor cores, which possibly included necrotic tissue. From our results, the dilation of small capillaries, related to tumor angiogenesis^85,87^, could be inferred in the GL261 periphery. In support of previous studies, we found that an elevated vessel tortuosity, even in singular, extreme cases, has the potential of serving as a geometric biomarker for tumor vasculature^61,85,88^.

Despite the disruptive effects of tumor growth on the local vasculature, the pathological vessel networks maintained basic classifications from graph theory that were also identified in healthy cerebrovascular networks, namely scale-free degree scaling with high exponents and the hierarchical clustering structure. This suggests that healthy and tumor-nurturing vascular networks both belong to the same class of transport networks with characteristic properties and topological scaling in size. Nonetheless, the uncovered network topology provides hints as to how vascular networks in the glioblastoma form. The dramatically increased relative abundance of terminal branches with degree *k* = 1 (vessel endpoints) in the tumor networks is a strong indicator for neovascularization^89^, pointing to sprouting angiogenesis, vascular mimicry, or vasculogenesis as likely mechanisms^90,91^. Elevated vessel tortuosities $\tau$ and local clustering coefficients *C* in the glioblastoma support the impression of angiogenesis playing an important role in the network formation, while promoting an increased abundance of vessel loops^86^. Not all signs point to angiogenesis, though; the decreased branching density *MVD* and longer vessel segments in tumor tissue are atypical for brain tumor angiogenesis^85^.

The breakdown in node diversity towards lower degree intersections, observed in tumor cores, indicates a degeneration of the preexisting vasculature during tumor development. This is supported by the significant decomposition of modular community sizes and general decrease in vascular density. Vessel occlusions on a large scale must have broken down the original, healthy network, eliminating high degree nodes and splitting existing vessel clusters. While tumor cores were supplied by sparse, very small and scarcely interconnected vessel communities, the periphery was found to maintain larger communities with more diversity and higher community clustering.

In glioblastoma networks, practically the same modularity was maintained by smaller community structures with stronger assortative mixing. Large supply entities were abandoned during tumor growth with focus on local metabolic needs and the effective transport of nutrients and oxygen. With small, light vessel clusters, nutrient rich blood can be transported long distances without being deprived along the way through large, dense community structures. The prospective gain in long-range transport efficiency leaves regions along the way undersupplied. The topological remodeling observed here may play an important role in the formation of hypoxic and necrotic regions and should be further investigated over a time course during tumor progression.

Albeit a decreased volume density of intercommunity vessel edges ${\rho }_{ice}$ at considerably higher density, ${\rho }_{c}$, of smaller communities, the clustered glioblastoma networks presented relatively short characteristic path lengths *L*_*c*_. The assortative mixing may be related to this aspect, which is, in effect, again geared towards the transport efficiency of the network. The small-world property has been investigated briefly on relatively large sections of the vibrissa primary sensory cortex of mice^30^. There, the absence of strict graph theoretical cliques was interpreted as an indicator that the studied microvasculature did not form small-world networks (cf.^30^, Online Methods). A reliable assessment of the small-world property should evaluate the scaling of the characteristic path length *L*_*c*_ with the number of meta-nodes *N*_*c*_^53^. This is not possible with, respectively, 6 samples in similar size ranges, but one should note the short mean path length of $\langle {L}_{c}\rangle$ ≈ 3–5, that separates most of the roughly 10^2^ communities in each network.

### Vasculature as a complex network

From a graph theoretical standpoint, the vascular networks quantified in this study present very unusual topological properties. Many factors can be involved in forming these networks, but a consensus in most theoretical models, treating the formation of scale-free networks, is that the dynamic growth process plays a central role in the emergence of a power law degree distribution. This is an important aspect in the original Barabási-Albert model^46^, as well as most methods thereafter, incorporating, *e*.*g*., preferential attachment, fitness models, and edge dynamics (for review, see, *e*.*g*.^53,69^).

Although recent years saw numerous publications on scale-free networks with degree exponents $\gamma \le 3$, scientific literature lacks the documentation and treatment of large, complex networks with high degree exponents, as encountered here. The similarity of *γ*-exponents in both types of networks suggests that the scale-free property with high degree exponents is immanent to large intracranial vessel networks, healthy and pathological, at the capillary scale. A previous study, modeling the Havers and Volkmann channels in cat humeri, revealed scale-free characteristics with degree exponents *γ* ≈ 3.7–3.8^92^. Although arguably in a different system, these vascular networks also present unusually high scaling exponents.

It has been shown that scale-free networks can be very resilient against random failures, since, if a fraction of nodes chosen randomly is lost, a majority of them is expected to have low degree. In random networks, highly connected vertices, often called hubs, are usually responsible for the global connectedness in the graph^53^. Thus, for random networks, a certain abundance of hubs is important for a system’s stability and some models have shown that exponents $\gamma  < 3$ result in increased robustness against random failures, while higher exponents lead to a quicker loss of global connectivity^53,93^. In the context of blood vessel networks, a node with high degree is not necessarily a node of central importance for nonlocal connectivity. Adapting the earlier argument, an increased exponent *γ* should result in a decreased likelihood of losing locally important high degree nodes from random failures. In this case, this speaks for a strong sustainability of the system’s diversity against stochastic damaging events.

The vascular networks exhibit relatively high clustering coefficients compared to random graphs. In the vascular context, high clustering coefficients manifest in local vessel loops consisting of only three edges, which guarantees high network stability, but, in abundance, is inefficient for nutrient and waste transport. The predominance of closed paths in intracranial vasculature, though mostly formed by a larger number of edges, has been shown to serve in flow rebalancing upon vessel occlusion^28,94^. Large vessel loops were not investigated in this study, but increased clustering coefficients in the pathological networks indicate that glioblastoma promotes good conditions for flow rebalancing and ensures local supply somewhat redundantly.

The vessel networks’ clustering with ${C}_{i}({k}_{i})\sim {k}_{i}^{-\beta }$ is reminiscent of hierarchical networks. Such scaling has been found in some real networks and can be reproduced by several models, incorporating different network evolution mechanisms^70,95^. Although it has been reported that the scaling exponent in true hierarchical networks often takes on values $\beta \approx 1$, and this has been proven analytically for two hierarchical network models^96,97^, deviating values still present the power-law scaling with degree. To our knowledge, hierarchical clustering has not been observed in real networks embedded in Euclidean space. It has been assumed, so far, that the spatial constraints, linked to cost factors in connectivity, suppress the formation of such hierarchical structures in spatial networks^70,73^.

The basic vascular networks combined scale-free degree distributions with hierarchical scaling of clustering coefficients, while strictly embedded in Euclidian space. The scale-free property supports the stability of the network over long time periods^93^, while the hierarchical organization can be expected to be related to the optimization of transport efficiency^7^. To our knowledge, no comparable real network with such high degree and clustering exponents, *γ* and *β*, has been quantified before. The findings suggest that tissue vasculature, when modeled as an undirected network, may form a distinct class of networks with unprecedented properties. Such a conclusion demands further investigations, including more statistics and different vascular networks, but the motivation for such studies should hereby be established.

### Methodological challenges

Intracranial vascular networks are composed of a myriad of individual vessel branches with different geometries. An attempt to study the full range of blood vessel instances pervading any animal tissue bears great experimental and computational challenges. The trade-off between high resolution and large acquisition volumes, that most imaging modalities are bound to, limits the capabilities to attain detailed and extensive anatomical information about full vascular systems. Regarding this compromise, the data acquisition enabling this study pushes the current frontiers of easily reproducible, large-scale biological imaging without need for co-registration or stitching.

The segmentation process plays a critical role in data treatment. Due to differently expressed imaging artefacts, each dataset was segmented individually with great care to reproduce the visual perception of vessels in the original image stacks. It may be argued that this produces subjective segmentations with no clear thresholds or fixed parameters between datasets, but, on the available data, the results are superior to alternative, threshold-based methods. Furthermore, any masking procedure used to differentiate healthy and tumor tissue suffers a certain ambiguity in tissue boundaries, which can influence statistical results.

It should be noted that the SPIM imaging procedure only incorporates perfused vessels. This is a positive feature in our context, since occluded vessels do not contribute to the network’s function, and are, a fortiori, not of interest in this study. However, a caveat regarding the presented geometric quantifications arises from the tissue clearing, where dehydration leads to an isotropic volume shrinkage of up to 40%^35^, which translates to vessel lengths and radii with a factor of ${0.6}^{1/3}\approx 0.84$. Since the smallest capillaries typically present *in vivo* diameters of around 4 *μ*m^98,99^, a shrinkage of 40% would correspond to a reduced vessel diameter of 3.37 *μ*m. In such extreme cases, with a resultion of 3.25–5 *μ*m in our study, more than 90% of the enclosing voxel will still be illuminated by vascular contrast, thus still safely registering the voxel as containing vasculature in accordance with Risser *et al*., who validated that a resolution in our range is just high enough to register the entire microvasculature^100^.

A highly accurate quantification for small capillaries was not possible in this study, as vessels with radii below approximately 3 *μ*m appear with single-voxel thickness. This pitfall was acceptable, as the aim of this paper was not to advance the large body of literature dealing with absolute geometric vessel properties, but to provide a detailed topological analysis of the entire microvasculature. The vascular network topology is not affected by tissue shrinkage or vascular radius distortion, since it only considers connections between vessels. One exception are distance-dependent measures, in which case physical separations are expected to scale linearly with a factor close to ${0.6}^{-1/3}\approx 1.186$ in the original tissue before clearing^35^. The vessel connectivity, however, is robust under the imaging and post-processing and the geometric properties are comparable within the scope of an experiment. The inclusion of the smallest vessels in our analysis was crucial to uncover the true network topology, as the capillary bed has been shown to have dense, mesh-like properties^5,18^.

A general issue with topological studies of biological data is the skeletonization process. Irregular surfaces and boundary perturbations cause single voxel stubs in the skeleton, which are by definition nodes with degree $k=2$^41^. Even though such nodes do not contribute to the vessel network in any sensible way, they were not removed from our analysis. Although pruning can help eliminate such nodes^41^, we refrained from such manipulations with arbitrary parameter choice in pruning length and method to avoid unnecessary data manipulation.

Another systematic effect of discrete image data is the emergence of high degree nodes. With high local node densities, neighboring branching points in the skeleton, *e*.*g*., consecutive bifurcations, can combine to vertices of high degree^41^. Examples are shown in Supplementary Movies 4 and 5. This effect produces long tails in the degree distributions, which, at first glance, may seem unphysiological. While higher resolution acquisitions are expected to break the high-degree nodes up into multiple low-degree branching points, the emerging power law in degree distributions is nonetheless meaningful at the treated length scale. From a large-scale perspective, branching points, which are separated by less than the diameter of the network’s smallest vessels, can sensibly be modeled as single meeting points of multiple vessels. When considered during the interpretation of the results, the implications of this caveat on our understanding of such large transport networks are rather constructive.

General topological properties of the cerebral vasculature have barely been quantified in the past. Although graph theoretical modeling has been applied to more and more anatomical systems in recent years^22,64^, our work provides the first multi-scale topological quantifications of the cerebral vasculature in a mammal to this detail. Furthermore, over the past decades, many studies have elucidated geometric and structural abnormalities of tumor vasculature in diverse settings and contexts^14,15,23^, but none have investigated the topological consequences of tumor growth on an entire vascular network.

The topological quantifications presented here only utilize a small subset of tools available in network theory to delineate the nature of complex networks. As the amount of data describing large systems and the availability of computational power have increased, a multitude of methods has been developed in the field of graph theory (see^59^ for a recent overview). Our quantifications of such large samples of the cerebral angiome are, to our knowledge, the first of this scale and detail. The undirected graph framework was employed in order to deliver first basic network characteristics. On such large systems made up of many similar constituents, this approach has proven to be successful in uncovering previously veiled system properties. Future studies should build on these results and extend our understanding of large vascular systems as complex networks, how tumor development alters these networks, and how we can use this in treatment.

## Conclusions

In conclusion, we found that tumor growth can alter the vascular topology without substantial reflections in geometric features of individual vessels in large-scale considerations. Tools from network theory are capable of grasping collective changes to the vascular network that are concealed in local analyses. This could better facilitate the delineation and grading of different forms of vascular remodeling, as demonstrated with the glioblastoma models U87 and GL261. The fundamental graph properties characterizing the cerebrovascular network were maintained in the glioblastoma, but local and nonlocal clustering, as well as long-range connectivity were characteristically rearranged, with more assortative mixing of strongly decomposed vessel communities. This may have profound implications on oxygenation and nutrient distribution to the tissue, which could be used for the development of tailored treatment strategies.

## Supplementary information

Supplementary Information

Supplementary Movie 1: Segmentation of a healthy brain hemisphere.

Supplementary Movie 2: Segmentation of a U87 glioblastoma.

Supplementary Movie 3: Segmentation of a GL261 glioblastoma.

Supplementary Movie 4: High degree node in healthy tissue.

Supplementary Movie 5: High degree node in U87 glioblastoma.
