# Supplementary material for: Glioblastoma multiforme restructures the topological connectivity of cerebrovascular networks
Source: Sci Rep. 2019 Aug 13;9:11757. doi: 10.1038/s41598-019-47567-w (PMC6692362; doi:10.1038/s41598-019-47567-w)
Supplement: Supplementary file 1 — Supplementary Information [file 41598_2019_47567_MOESM1_ESM.pdf]

Supplementary Information

Glioblastoma multiforme restructures the topological connectivity of cerebrovascular networks

Artur Hahn, Julia Bode, Thomas Krüwel, Gergely Solecki, Sabine Heiland, Martin Bendszus, Björn Tews, Frank Winkler, Michael O. Breckwoldt, and Felix T. Kurz

Supplementary Video Legends

Supplementary Movie 1: Segmentation of a healthy brain hemisphere. Segmentation results from a healthy mouse brain hemisphere, presenting raw imaging data from SPIM, overlaid with the binary vessel segmentation from ilastik in semitransparent red. The AVI-movie (.avi) shows 300 consecutive image slices, each 5 µm apart, at 25 frames per second (fps).

Supplementary Movie 2: Segmentation of a U87 glioblastoma. Segmentation results from a U87 glioblastoma specimen in a mouse brain, presenting raw SPIM data, overlaid with the binary vessel segmentation from ilastik in semitransparent red. The AVI-movie shows 500 consecutive image slices, each 5 µm apart, at 25 fps.

Supplementary Movie 3: Segmentation of a GL261 glioblastoma. Segmentation results from a GL261 glioblastoma xenograft, with raw SPIM data, overlaid with the ilastik segmentation in semitransparent red. The AVI-movie shows 400 consecutive image slices, each 5 µm apart, at 25 fps.

Supplementary Movie 4: High degree node in healthy tissue. AVI-movie, showing a volume rendering of a vascular subvolume from healthy grey matter, comprising a volume of 130 x 130 x 100 µm, depicting a node with degree k=24 (extended node voxels marked in glowing orange). The first 360° rotation shows the segmented and post-processed vasculature in semitransparent red, followed by the additional appearance of the vascular skeleton voxels, as used for analysis, in blue for another 360° rotation. For the last rotation, the discrete skeleton representation is replaced by tube structures, determined in the image processing software Amira 5.4.1 (Thermo Fisher Scientific, Waltham, MA, USA) for better visualization. The movie frames were created using Amira ResolveRT FEI 5.4.

Supplementary Movie 5: High degree node in U87 glioblastoma. AVI-movie showing a volume rendering of a vascular subvolume from a U87 glioblastoma's peripheral region, comprising a volume of 188 x 188 x 110 µm, depicting a node with degree k=14 (extended node voxels marked in glowing orange). The first 360° rotation shows the segmented and post-processed vasculature in semitransparent red, followed by the additional appearance of the vascular skeleton voxels, as used for analysis, in blue for another 360° rotation. For the last rotation, the discrete skeleton representation is replaced by tube structures, determined in the image processing software Amira for better visualization. The movie frames were created using Amira.
